# HBcAb Positivity as a Risk Factor for Missing HIV RNA Undetectability after the 3TC+DTG Switch

**DOI:** 10.3390/v16030348

**Published:** 2024-02-23

**Authors:** Vincenzo Malagnino, Tiziana Mulas, Elisabetta Teti, Monica Basso, Mario Giobbia, Nicholas Geremia, Giuliana Battagin, Yasmine Abi Aad, Jean-Paul Vincensini, Marco Iannetta, Saverio Giuseppe Parisi, Loredana Sarmati, Karine Lacombe

**Affiliations:** 1 Infectious Disease Unit, Policlinico Tor Vergata of Rome, 00133 Rome, Italy; tizia.mulas@gmail.com (T.M.); elisabetta.teti@gmail.com (E.T.); marco.iannetta@uniroma2.it (M.I.); sarmati@med.uniroma2.it (L.S.); 2 Department of System Medicine, Tor Vergata University of Rome, 00133 Rome, Italy; 3 Department of Molecular Medicine, University of Padova, 35128 Padova, Italy; monica.basso@unipd.it (M.B.); saverio.parisi@unipd.it (S.G.P.); 4 Infectious Disease Unit, Ospedale di Treviso, 31100 Treviso, Italy; mario.giobbia@aulss2.veneto.it; 5 Infectious Disease Unit, Ospedale di Venezia, 30122 Venezia, Italy; nicholas.geremia@aulss3.veneto.it; 6 Infectious Disease Unit, Ospedale di Vicenza, 36100 Vicenza, Italy; giuliana.battagin@aulss8.veneto.it; 7 Hôpital Saint-Antoine, Assistance Publique Des Hôpitaux de Paris, Service Des Maladies Infectieuses Et Tropicales, Cedex 12, 75571 Paris, France; yasmine.abiaad@aphp.fr (Y.A.A.); jpvincensini@gmail.com (J.-P.V.); karine.lacombe2@aphp.fr (K.L.); 8 INSERM, Pierre Louis Institute of Epidemiology and Public Health (IPLESP), Sorbonne University, 75646 Paris, France

**Keywords:** HBcAb+, OBI, HIV/HBV

## Abstract

Hepatitis B Core antibody (HBcAb) positivity is the surrogate marker of hepatitis B occult infection. This condition is not a contraindication for switching to two-drug (2DR) antiretroviral therapy; however, the removal of tenofovir may contribute to poor control of HBV replication. A multicentre retrospective cohort study investigated the impact of HBcAb positivity on HIV control in patients switching to a 2DR with Lamivudine and Dolutegravir (3TC-DTG). In this study, a comparison analysis was conducted between HBcAb-positive and -negative PLWH regarding HIV-RNA suppression, considering: (1): Target Not Detected (TND) < 20 cp/mL; (2) Target Detected (TD) < 20 cp/mL; and (3) Detectable > 20 cp/mL and <50 cp/mL and >50 copies/mL. A total of 267 patients on 2DR with 3TC-DTG were included. In comparison to HBcAb-negative, HBcAb-positive patients were older (45 years [35–54]) and had a lower CD4+ nadir (248 vs. 349 cells/mmc, *p* = 0.007). No difference in the maintenance of virological suppression was present in the two groups of patients before the switch. Although no patient had an HIV-RNA > 20 cp/mL after the switch, significantly fewer HBcAb-positive compared with -negative subjects resulted in TND at 12, 24, and 36 months after the switch: 52 (69.3%) versus 164 (85.4%), *p* = 0.004, 50 [72.5%] versus 143 [89.9%], *p* = 0.001, and 30 [66.7%] versus 90 [92.8%], *p* = 0.001, respectively. HBcAb positivity is associated with an increased risk of suboptimal HIV suppression during the 36 months after 3TC/DTG simplification. This finding reinforces the relevance of the OBI condition in PLWH and raises the issue of careful virological monitoring of such cases.

## 1. Introduction

Antiretroviral treatment (ART) simplification with two-drug regimens (2DRs) is a strategy that may reduce drug-induced toxicity and drug–drug interactions in older patients living with HIV (PLWH) and also prevent drug-related toxicity (pre-emptive switch) in young PLWH [1]. There is robust evidence regarding the efficacy of 2DRs in maintaining a suppressed HIV viremia [2], even in terms of residual viremia [3], whereas it is less clear if 2DRs maintain the same efficacy of triple ART in improving immunological recovery and reducing immune activation [4]. Altogether, knowledge regarding 2DR treatment is reassuring, and the potential benefits in tolerability, long-term toxicities, drug interactions, cost-effectiveness, patients’ compliance, and quality of life appear to outweigh the potential risks of reducing ART class composition [5].

In PLWH with Hepatitis B (HBV) S antigen (HBsAg) positivity, switching to 2DR (Lamivudine [3TC]-containing or nucleoside/nucleotide analogue [NUC]-sparing) is not recommended [6,7] both because of the high risk of severe hepatitis flares and decompensation following HBV reactivation, and because of the lower 3TC genetic barrier and the risk of a breakthrough due to HBV strains with archived YMDDs [8,9] and vaccine-escape-associated mutations [10].

HBcAb positivity (without the HBs antigen; regardless of the anti-HBs antibody status) is the surrogate marker of the Occult Hepatitis B Infection (OBI) condition [11] in immunocompromised patients. OBI has been associated with an increased risk of progression of liver fibrosis, cirrhosis, and the development of hepatocarcinoma in PLWH [12,13,14]. Salpini and colleagues recently showed that cryptic HBV replication (measured by highly sensitive digital-droplet PCR) is present in almost 30% of ART-treated HBcAb-positive PLWH and correlates with detectable HIV-RNA [15]. Cryptic HBV replication could explain the progression of liver disease in patients with HIV with OBI and underlie the poor control of HIV viraemia in HBcAb-positive PLWH after the introduction of first-line antiretroviral therapy (cART), as already demonstrated in other studies [16]. Low levels of HBV-DNA in immunocompromised subjects with OBI could be the expression of the transcriptional activity of the covalently closed circular DNA (cccDNA) present at the hepatic level [11]. In the setting of HIV infection, a low lymphocyte count could favour a less efficient control of HBV cryptic replication in patients with the OBI condition [13].

The finding of persistent low-level HIV viraemia events (between 50 and 1000 copies/mL) in patients on stable ART is common, and its occurrence has been associated with a higher likelihood of virologic failure, the emergence of resistance to drugs, and the evolution to AIDS [17]. Low-level viremia and residual viremia (detectable virus below 50 or 20 copies/mL) were also related to a persistent state of immune activation [18], a condition correlated with an increased risk of comorbidities in PLWH. A recent study [19] demonstrated that detectable viraemia of less than 20 copies/mL is present in over 40% of ART patients. This phenomenon is associated with an increased risk of viraemia > 20 copies in subsequent measurements, inversely correlated with the duration of virologic suppression while on therapy, and positively correlated with prior discontinuation of therapy. The study also demonstrated a positive effect of integrase inhibitor therapy, especially Dolutegravir (DTG), in maintaining undetectable viraemia < 20 copies/mL.

HBcAb positivity in the absence of HBs Ag does not actually represent a contraindication to therapeutic simplification to 2DRs, even if European guidelines recommend monitoring, for a possible risk of HBV reactivation, of HBcAb-positive PLWH who undergo immunosuppressive treatment for solid tumours or onco-haematological diseases [6].

The aim of this study was to evaluate the impact of HBcAb positivity in PLWH who have a negative HBsAg serology and undergo treatment simplification with a regimen containing Lamivudine and DTG co-formulated or not (3TC/DTG).

## 2. Materials and Methods

### 2.1. Study Design

A multicentre retrospective cohort study was conducted in 267 HIV—HBs antigen-negative patients undergoing a switch to 2DRs consisting of 3TC+DTG or a co-formulation of 3TC/DTG. Patients were enrolled at different clinical sites: the Infectious Diseases Clinic of the Tor Vergata Polyclinic in Rome, the Department of Molecular Medicine of the University of Padua, the Units of Infectious Diseases of Vicenza, Venezia and Treviso hospitals, and the Infectious Diseases Department of the Saint Antoine Hospital in Paris Sorbonne University. For this study, a database was built, collecting the following data for all patients: demographic information, first HBV serology available after the HIV diagnosis, CD4+ cell count at HIV diagnosis and at the time of the 2DR switch, calendar year of HIV infection diagnosis, ART composition before the 2DR switch, and HIV-RNA viral load at 6, 12, 18, and 24 months (±1 month) before the 2DR switch, at the time of the switch (considered baseline of the study), and at 6, 12, 24, 36, and 48 months (±1 month) after.

### 2.2. Inclusion and Exclusion Criteria

**Figure 1:**
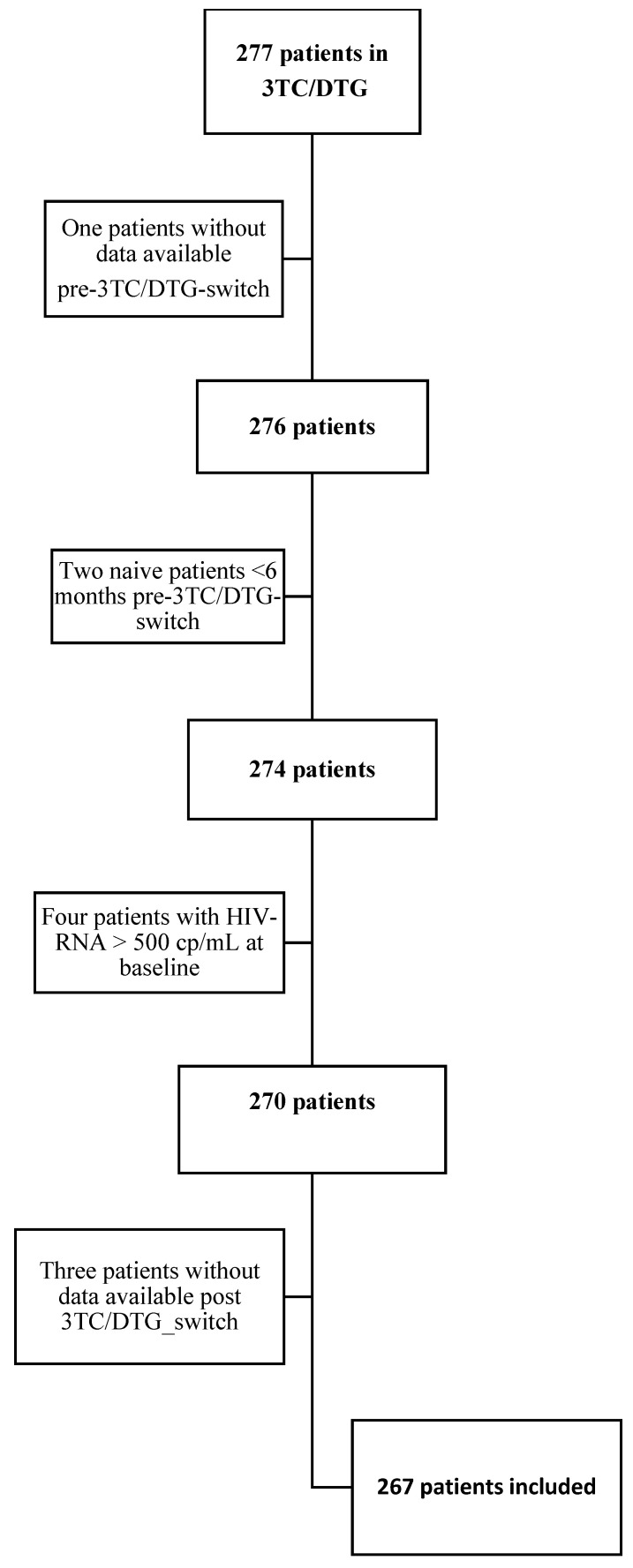
Patient inclusion and exclusion algorithm.

The patient inclusion and exclusion algorithm is shown in Figure 1. All patients undergoing a switch to dual therapy containing 3TC+DTG or 3TC/DTG from the different centres were included. Specific inclusion criteria were as follows: virology suppression with HIV-RNA < 50 cp/mL at baseline and availability of follow-up data as described above. As described in Figure 1, 10 of 277 patients on a 2DR were excluded from the study population because of the unavailability of immunovirological information at pre-switch visits (n = 1), less than one year of ARV exposure before the switch (n = 2), an HIV-RNA value > 500 cp/mL at baseline (n = 4), or unavailable post-switch data (either due to loss to follow-up or unscheduled visits within the required time frame, n = 3).

### 2.3. Laboratory Testing for the Diagnosis of HBV and HIV Infections

The qualitative detection of HBcAb, HBsAb, and HBsAg serological markers was performed by using immune-enzymatic assays (Roche/Cobas Diagnostics, Rotkreuz, Switzerland). Plasma HIV-RNA levels were measured using a commercial test characterized by a lower limit of quantification (LLOQ) for HIV-RNA of 20 copies/mL COBAS AmpliPrep/COBAS TaqMan HIV-1 Test, v2.0). The test allows for the detection and also the presence of HIV-RNA < 20 HIV-RNA copies/mL, a condition that is defined as HIV-RNA detectability below the LLOQ of the assay. HCV-RNA quantification was performed by using the COBAS^®^-AmpliPrep/COBAS^®^-TaqMan^®^ HCV Qualitative Test, v2.0 (LLOD = LLOQ = 15 IU/mL; Roche Molecular Systems Inc., Pleasanton, CA, USA), and antibody anti-HCV was performed using the Alinity i chemioluminescence assay (Abbott, Abbott Park, IL, USA).

### 2.4. HIV Viral Load Definitions

To evaluate HIV viremia kinetics after the 2DR switch in the study cohort, all HIV viral load measurements were carried out at 6, 12, and, if available, 24 months before and at 6, 12, 24, 36, and 48 months after the transition to the 2DR were considered. Based on the results of HIV-RNA obtained by the specified commercial Real-Time assay, the following definitions were adopted: (1): HIV-RNA < 20 cp/mL Target Not Detected (TND); (2) HIV-RNA < 20 cp/mL Target Detected (TD); and (3) 20 cp/mL < HIV-RNA Detectable < 50 cp/mL; and (4) HIV-RNA > 50 cp/mL. Viremia values higher than 20 copies/mL and the detection of HIV-RNA below the LLOQ of 20 copies/mL were considered signs of active HIV replication.

### 2.5. Endpoints

The primary endpoint of this study was to evaluate differences in the maintenance of HIV viremia suppression < 20 copies/mL between the two PLWH populations of HBcAb-positive and HBcAb-negative patients at 6, 12, 24, and 36 months after the 2DR 3TC/DTG-based switch.

### 2.6. Statistical Methods

Data were collected, and the dataset was assembled using Excel 2019 version 16.3. The study population is described using proportions and percentages for categorical values, median measurements, and interquartile ranges (IQRs) for continuous values. The comparison between PLWH-HBcAb-positive and PLWH-HBcAb-negative patients was performed with the Kruskal–Wallis test for continuous variables and with the chi-squared test or Fisher’s exact test, when appropriate, for categorical variables. Univariable odds ratios (ORs) associated with a lack of HIV RNA undetectability at T24 and T48 and their 95% confidence intervals (CIs) were calculated using logistic regression. A multivariable model was constructed in which risk factors with *p* < 0.1 in the univariable analysis were included in a full model, which were then excluded in a backwards-stepwise fashion if *p* < 0.05 using the likelihood ratio test. All statistical analyses were conducted using STATA 14.2 (College Station, TX, USA).

### 2.7. Ethic Statements

The study protocol and related informed consent were submitted and approved by the Independent Ethics Committee at Policlinico Tor Vergata [Protocol Number 216/16, version 1.0] for the Italian centres. All included patients signed an informed consent for inclusion in the observation. For France, this research falls within the framework of the “Reference Methodology for research not involving the human person, studies and evaluations in the field of health” (MR004). The Infectious and Tropical Diseases Department of Saint-Antoine Hospital signed a commitment to comply with this “Reference Methodology”. Only people who gave their written consent to use their data for research purposes were included in this study.

## 3. Results

### 3.1. General Population

Table 1 summarizes the characteristics of the 267 patients included in this study. The majority were male (181/86, 67.8%) with a median age of 41 years (interquartile range IQR 32–52). On average, the patients had a 10-year history of HIV infection (calendar year of HIV diagnosis 2012 [IQR 2005–2014]). Serological tests for co-infection with hepatotropic viruses identified 76 (28.4%) HBcAb-positive patients, of whom 56 (74.7%) had HBsAb-protective antibodies, and 34 (13.3%) HCVAb-positive, all negative for HCV-RNA. In general, the study population experienced a gradual recovery of CD4+ lymphocytes: median CD4+ nadir 306 (IQR 203–442) versus a median CD4+ cell count at the switch of 754 cells/mmc (IQR 562–961). All enrolled patients had an HIV-RNA value below the 50 cp/mL threshold and 254 (95.1%) were <20 cp/mL (HIV-RNA TND or TD). A good virological control was also observed at the different time points before the switch to 3TC/DTG. At 6, 12, and 24 months before the switch, all patients with available data had HIV-RNA < 50 cp/mL, and at least 85% of the patients had HIV-RNA < 20 cp/mL. Encouragingly, the proportion of patients with detectable viremia between 20 and 50 cp/mL was decreasing at the different timepoints prior to the switch: 13.1% of patients at 24 months, 12.4% at 12 months, 8.2% at 6 months, and 4.9% at the time of the switch.

It is noteworthy that all included patients had HIV RNA below 50 cp/mL at the time of the switch. Moreover, more than 95% of patients (254 [95.1%]) showed optimal HIV suppression at the time of the switch to 3TC/DTG, with HIV-RNA below 20 cp/mL, although of these, 228 (85.4%) with HIVcompletely undetectable and 26 (9.7%) with HIV-RNA detectable under 20 cp/mL. We can assume that the patient population included at the time of the switch was in optimal virological control of HIV infection undergoing 3DR antiretroviral therapy.

### 3.2. HIV-RNA Control after the Switch to 2DR Therapy

HIV-RNA data were collected at 6, 12, 24, and 36 months after the switch to 2DRs, and the data show a general efficacy in maintaining HIV-RNA <50 cp/mL (Table 1). The evolution of the proportions of patients < 50 cp/mL, TD, and TND are reported in Figure 1. Overall, there was an observed progressive increase in the proportion of patients with detectable HIV-RNA after the switch to a 2DR. Furthermore, none of the 22 (8.2%) patients who were TD at 6 months after switching to a 2DR had viraemia values greater than 50 cp/mL. Of the 23 patients (9.2%) with viremia > 50 cp/mL 12 months after the switch, 9 (3.6%) had a viremia between 50 and 200 cp/mL. Of the 14 (6.2%) patients with viremia > 50 cp/mL 24 months after the switch, 4 (1.8%) had a viraemia between 50 and 200 cp/mL. Finally, 36 months after the switch, out of five patients (3.5%) with viraemia > 50 cp/mL, one (0.7%) had 136 cp/mL of HIV-RNA.

Overall, among patients with available data who maintained HIV-RNA < 20 copies/mL, the total number of patients with a constant TND status was 149 (66.8%) at 24 months and 89 (50.5%) at 36 months.

### 3.3. Comparison between HBcAb-Positive and HBcAb-Negative Patients

The comparison between HBcAb-positive and -negative patient characteristics is reported in Table 2. HBcAb-positive patients were older than HBcAb-negative patients (45 years [IQR 35–54] versus 40 [32–50], *p* = 0.015), whereas CD4+ lymphocyte nadir was lower in HBcAb-positive compared with HBcAb-negative patients (248 cells/mmc [IQR 153–388] versus 349 [IQR 214–463], *p* = 0.007), However, full immune recovery was observed during ART, with a CD4+ cell value at the time of the switch to a 2DR similar in the two groups (767 [IQR 557–994] versus 741 [IQR 566–958], *p* = 0.84). HBcAb-positive patients were more frequently HCV-antibody-positive (16 [21.3%] versus 18 [9.8%] *p* = 0.013), but all were HCV-RNA-negative.

**Figure 2:**
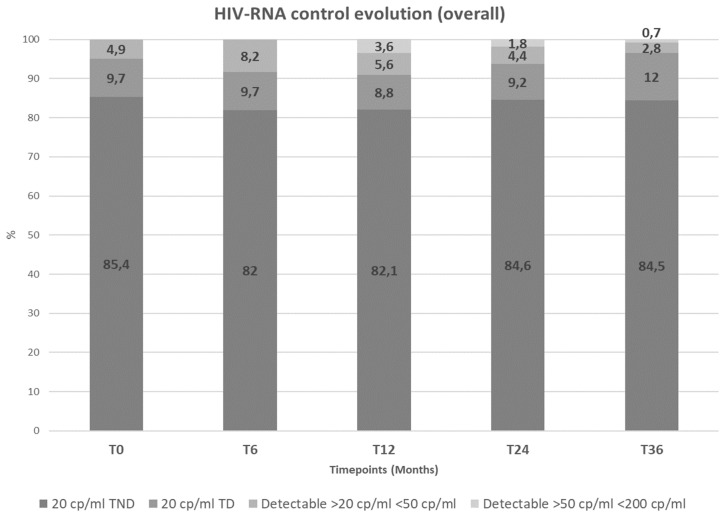
HIV-RNA control during follow-up in the general population.

**Figure 3:**
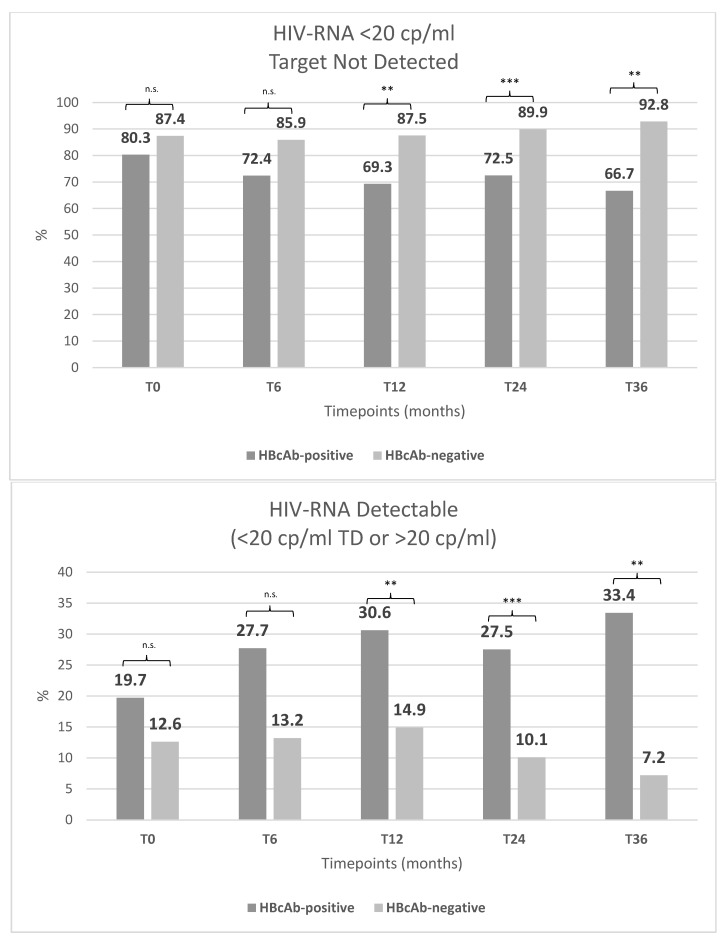
Trends in HIV-RNA control during follow-up in different subpopulations. Abbreviations: n.s.: not significance; ** *p*-value < 0.001; *** *p*-value < 0.0001.

Figure 2 reports the evolution of the proportion of patients with TD and TND status over time according to HBcAb status. During follow-up at different timepoints (Figure 3), no difference between TND and TD status was demonstrated in the two groups at 6 (*p* = 0.15), 12 (*p* = 0.30), or 24 (*p* = 0.15) months before the switch. Six months after the switch, the proportion of HBcAb-positive patients with TND status was lower than that of HBcAb-negative patients (72.4% vs. 85.9%, respectively, (*p* = 0.058).

Twelve months after the switch, a significantly lower number of HBcAb-positive subjects were TND (52 [69.3%] vs. 154 [87.5%], *p* = 0.004). Again, at 12 months post-switch, in the group of HBcAb-positive patients, there was a significantly greater number of TD subjects (13 [17.3%] vs. 9 [5.1%], respectively, in the HBcAb-positive and -negative groups) and with detectable viraemia between 20 and 50 copies/mL (6 [8%] vs. 8 [4.5%], respectively, in the HBcAb-positive and -negative groups) (*p* = 0.004).

Similar results were obtained at 24 and 36 months after the switch, with a higher proportion of subjects with TD below or between 20 and 50 copies/mL in the HBc-positive group compared with the -negative group (at 24 months: 15 [21.7%] versus 6 [3.8%], and 4 [5.8%] vs. 6 [3.8%], respectively, [*p* < 0.0001]; at 36 months: 12 [26.7%] vs. 5 [5.1%] and 2 [4.5%] vs. 2 [2.1%], respectively [*p* = 0.001]).

The rate of patients found to be consistently TND during all study timepoints at 24 and 36 months after the 3TC/DTG switch was investigated. At 24 months, half of PLWH/HBcAb-positive patients with available data were TND at all timepoints, whereas 115 (74.2%) PLWH/HBcAb-negative patients maintained optimal HIV suppression during the 2DR (*p* < 0.0001). Nevertheless, at 36 months after the switch, only 16 (18%) PLWH/HBcAb-positive patients, as opposed to more than half (73 [59.9%]), achieved complete virological suppression while taking a 2DR. This analysis shows a suboptimal HIV-RNA suppression during 3TC/DTG that is much more evident in PLWH/HBcAb-positive patients.

A multivariate analysis was conducted to assess risk factors predictive of HIV virological detectability at 36 months in the study population (Table 3). Age, calendar year of infection, CD4+ cell count nadir, ART virological failure, and HBcAb status were considered. The presence of HBcAb-positivity was the only variable associated with HIV-RNA detectability (OR 3.07 [CI95% 1.39–6.79], *p* = 0.005).

## 4. Discussion

The results of our study demonstrated that the risk of developing HIV viremia detectability (<20 cp/mL TD or >20 cp/mL) after the switch to a 2DR containing 3TC-DTG was multiplied by approximately 3 in HBcAb-positive PLWH. Incomplete HIV suppression appeared in a progressively increasing proportion of HBcAb-positive subjects as early as 12 months, and then at all timepoints up to 36 months after the switch.

The persistence of low-level HIV viraemia (>50 copies/mL), and also residual viraemia (TND < 20 and detectable viremia between 20–50 copies/mL), has been correlated to virological failure and the possible emergence of drug resistance [17]. In a population of 1214 ART-treated patients, Maggiolo et al. demonstrated that even HIV viraemia as low as three copies per millilitre is linked to a significantly increased risk of virological failure and genotypic resistance [20].

The causes of low-level or residual HIV viraemia in ART-treated patients are not fully understood to date. A large latent reservoir and advanced HIV infection at ART initiation, as well as poor potency of ART or suboptimal adherence to therapy, are all possible causes that have been associated with low-level HIV replication during ART [19]. Moreover, coinfections (i.e., HBV, Hepatitis C virus, tuberculosis, syphilis) may contribute to increased inflammation that could fuel HIV replication [21]. In a previous study from an Italian multicentre cohort where 2DR-treated patients were followed for 48 weeks, a significantly reduced proportion of HBcAb-positive compared with HBcAb-negative PLWHs remained TND (3.5% versus 50.7%, *p* < 0.0001) [22], despite the dual treatments all containing 3TC in combination with protease inhibitors (Atazanavir and Darunavir) or DTG. Salpini and colleagues recently found that more than 30% of patients on TDF/TAF containing ART have detectable HBV-DNA measured by highly sensitive digital droplet PCR.

Moreover, after TDF/TAF withdrawal, HBV reactivation occurred in 40 (39.6%) patients, 32.5% of whom developed HBV-DNA >10 IU/mL, and 25% had an increase in transaminase level; close correlation between the presence of low-copy HBV replication (cryptic HBV replication) and the simultaneous presence of detectable plasma HIV-RNA has been previously demonstrated [15]. Several publications [23] reported the proportion of OBI infection in PLWH groups, overall ranging between 15 and 30%, and detectability of HBV-DNA (>10 UI/mL) has been observed in 0.6–2.5% of HBcAb PLWH. The appearance and increase in HBV-DNA in HBcAb-positive PLWH is associated with immunosuppressive drug use, chemotherapy, and treatment of hepatitis C, and also with the withdrawal of HBV-active drugs [24,25,26]. Moreover, Coffin and colleagues underlined residual HBV replication in PLWH/HBcAb-positive in lamivudine-containing long-term cART that can promote the emergence of YMDD mutations in reverse transcriptase (RT) associated with lamivudine resistance [27]. This can explain the limited impact and protection of 3TC, with a consequent increase in HBV cryptic replication.

The mechanisms by which HBV replication can regulate HIV-1 transcription are still largely unknown, although there is some evidence in the literature concerning the role of HBx in the regulation of HIV-1 replication. Mu and colleagues [28] found that the alteration of proteins C/EBP and CREB recognition elements inhibited but did not abolish the activation of HIV-1 LTR regulated by HBx; they also identified that C/EBP and CREB recognition elements within the LTR-129 to -80 regions of HIV-1 LTR were important for HIV-1 LTR transcription in response to HBx regulation. Although not analysed in the literature, our hypothesis identifies the above-mentioned mechanism of transcriptional transactivation of HIV by HBV, even in the absence of HBsAg, as in our cohort, favouring residual or low-level viraemia in patients undergoing therapeutic switch by sacrificing highly active anti-HBV drugs.

Regarding the management of PLWH with the serological signs of OBI, the British HIV Association (BHIVA) guidelines for the management of coinfection with HIV-1 and hepatitis B or C virus do not give indications on treatment strategies for HBcAb-positive PLWH [29], while the European AIDS Clinical Society (EACS) suggests the maintenance of anti-HBV active treatment only in HBc-positive patients receiving immunosuppressive therapy or a switch to Entecavir (ETV) if TAF/TDF is contraindicated [30]. European guidelines in particular emphasize caution when switching from a TDF/TAF-based regimen to drugs with a lower genetic barrier (Emtricitabine [FTC] or 3TC), and persons with HIV with isolated anti-HBc concerning viral breakthrough or relapse of HBV. Furthermore, a transaminase and HBV-DNA check is regularly recommended during follow-up.

Clinical trials on PLWH suppressed and switched to 3TC/DTG [31,32] excluded HBsAg-negative patients and HBcAb-positive patients with detectable HBV-DNA, but did not exclude HBcAb-positive patients with HBsAb, considering them immune to HBV infection [33,34]. The same criteria were used in different settings, in the GEMINI 1 and 2 trials on naive PLWH starting 3TC/DTG [34]. On this issue, in the above-mentioned study conducted by Salpini and colleagues, in the cohort of 94 PLWH/HBcAb+ with available data for HBsAb titre, as many as 63 (67%) were positive for protective antibody titre, assuming an impact of HBsAb titre on the control of cryptic HBV-DNA viremia in liver transplant patients and HIV [35,36]. For this reason, a further analysis was conducted comparing PLWH/HBcAb+ with and without HBsAb, but no statistically significant difference was found between the subgroups in terms of HIV viraemia suppression after the 3TC/DTG switch. Also, in light of previous studies that investigated the HBV vaccination response in PLWH with isolated HBcAb positivity [37,38,39], prospective studies correlating the response in terms of HIV and HBV suppression rates in this category of patients after HBV vaccination, maybe with a reinforced schedule, are to be considered.

Our study has some limitations to acknowledge. First, the retrospective nature of the data collection did not allow for the control of certain characteristics of the included patients such as transaminase measurements and liver fibrosis evolution during follow-up, and certain anamnestic characteristics such as alcohol intake and intravenous drug use were not included in this study for the same reason. Furthermore, it did not allow for the systematic collection of HBV-DNA data concomitant to immunovirological data for HIV infection. Considering the data collected and the limitations of this study, a prospective study with periodic HBV-DNA monitoring, perhaps by ddPCR methods and the use of new HBV serological markers, such as HBV-RNA and HBcAb quantitative titre, is desirable to stratify the risk of HIV suboptimal suppression and HBV rebound during the simplification of antiretroviral therapy with TDF/TAF-sparing regimens in PLWH/HBcAb-positive patients. Since it is not systematically provided for in the clinical practice of all centres, it was not possible to include data on adherence to antiretroviral treatment, although virological success with HIV-RNA subthreshold detection was considered in the inclusion criteria and used as a surrogate for the adherence to therapy.

To the best of our knowledge, this is the first study to analyse the impact of HBCAb positivity on the control of HIV virological suppression during the therapeutic switch to 3TC-DTG. HbcAb positivity in PLWH without the HBs antigen seems to represent a risk factor for suboptimal HIV suppression in 2DRs that needs to be taken into account in the strategy of therapeutic simplification with no active or suboptimal drug against HBV. This is particularly important given the increased use of long-acting (LA) strategies. Further studies with prospective designs are needed to confirm these results.

## Figures and Tables

**Table 1:** Characteristics of study population.

		N = 267
Sex ratio M/F, %F		181/86 (32.2%)
Age, years		41 (32–52)
Calendar year of HIV diagnosis, median		2012 (2005–2014)
Nadir CD4+, cell/mmc		306 (203–442)
cART pre-switch, n (%):		
	2NRTI+PI	41 (15.4%)
	2NRTI+NNRTI	89 (33.3%)
	2NRTI+INI	125 (46.8%)
	Other	12 (4.5%)
CD4+ at 2DR switch, cell/mmc		754 (562–961)
HCVAb+ serology, n (%)		34 (13.3%)
Previous HIV viral rebound, n (%)		32 (16.9%)
HIV diagnosis < 12 months before 3TC/DTG switch, n (%)		9 (3.4%)
HIV diagnosis < 24 months before 3TC/DTG switch, n (%)		10 (3.75%)
HBcAb+, n (%)		76 (28.4%)
HBcAb+/HBsAb+, pts, n (%)		56 (74.7%)
HIV RNA 24 months pre-2DR switch, n (%):		
	<20 cp/mL TND	181 (74.2%)
	<20 cp/mL TD	31 (12.7%)
	Detectable > 20 cp/mL <50 cp/mL	32 (13.1%)
HIV RNA 12 months pre-2DR switch, n (%):		
	<20 cp/mL TND	219 (82.3%)
	<20 cp/mL TD	14 (5.3%)
	Detectable > 20 cp/mL <50 cp/mL	33 (12.4%)
HIV RNA 6 months pre-2DR switch, n (%):		
	<20 cp/mL TND	225 (87.5%)
	<20 cp/mL TD	11 (4.3%)
	Detectable > 20 cp/mL <50 cp/mL	21 (8.2%)
HIV RNA at 2DR switch, n (%):		
	<20 cp/mL TND	228 (85.4%)
	<20 cp/mL TD	26 (9.7%)
	Detectable > 20 cp/mL <50 cp/mL	13 (4.9%)
HIV RNA 6 months post-2DR switch, n (%):		
	<20 cp/mL TND	219 (82%)
	<20 cp/mL TD	26 (9.7%)
	Detectable > 20 cp/mL <50 cp/mL	22 (8.2%)
HIV RNA 12 months post-2DR switch, n (%) *:		
	<20 cp/mL TND	206 (82.1%)
	<20 cp/mL TD	22 (8.8%)
	Detectable > 20 cp/mL <50 cp/mL	14 (5.6%)
	Detectable > 50 cp/mL <200 cp/mL	9 (3.6%)
HIV RNA 24 months post-2DR switch, n (%) **:		
	<20 cp/mL TND	193 (84.6%)
	<20 cp/mL TD	21 (9.2%)
	Detectable > 20 cp/mL <50 cp/mL	10 (4.4%)
	Detectable > 50 cp/mL <200 cp/mL	4 (1.8%)
HIV RNA 36 months post-2DR switch, n (%) ***:		
	<20 cp/mL TND	120 (84.5%)
	<20 cp/mL TD	17 (12%)
	Detectable > 20 cp/mL <50 cp/mL	4 (2.8%)
	Detectable > 50 cp/mL <200 cp/mL	1 (0.7%)
HIV RNA 48 months post-2DR switch, n (%) #:		
	<20 cp/mL TND	61 (76.2%)
	<20 cp/mL TD	13 (16.2%)
	Detectable > 20 cp/mL <50 cp/mL	4 (5%)
	Detectable > 50 cp/mL <200 cp/mL	2 (2.5%)
TND during 36 months, n (%)°°		89 (50.5%)

* data available for 251 pts; ** data available for 228 pts; *** data available for 142 pts; # data available for 80 pts; °° data available for 176 pts; Abbr: 2DR: two-drug regimen; NRTI: Nucleoside Reverse Transcriptase Inhibitor; NNRTI: Non-Nucleoside Reverse Transcriptase Inhibitor; PI: Protease Inhibitor; INI: Integrase Inhibitor; 3TC: Lamivudine; DTG: Dolutegravir; TND: Target Not Detected; TD: Target Detected.

**Table 2:** Comparison of AntiHBc + vs. AntiHBc- subpopulations.

		HBcAb+ (n = 76)	HBcAb- (n = 191)	*p*-Value
Sex ratio M/F, %F		49/27 (35.5%)	132/59 (30.9%)	0.46
Age, years		45 (35–54)	40 (32–50)	**0.015**
Calendar year of HIV diagnosis, median		2011 (2005–2013)	2012 (2007–2014)	0.11
Nadir CD4+, cell/mmc		248 (153–388)	349 (214–463)	**0.007**
Hystory of HIV viral rebound, n (%)		13 (25.5%)	19 (13.7%)	0.056
cART pre-switch, n (%):				0.32
	2NRTI+PI	14 (18.4%)	27 (14.1%)	
	2NRTI+NNRTI	26 (34.2%)	63 (33%)	
	2NRTI+INI	30 (39.5%)	95 (49.7%)	
	Other	6 (3.1%)	6 (7.9%)	
CD4+ at 2DR switch, cell/mmc		767 (557–994)	741 (566–958)	0.84
HCVAb+ serology, n (%)		16 (21.3%)	18 (9.8%)	**0.013**
HIV diagnosis < 12 months before 3TC/DTG switch, n (%)		2 (2.6%)	8 (4.2%)	0.54
HIV diagnosis < 24 months before 3TC/DTG switch, n (%)		3 (3.9%)	6 (3.1%)	0.74
HIV RNA 24 months pre-2DR switch, n (%):				0.35
	<20 cp/mL TND	49 (68.1%)	132 (76.7%)	
	<20 cp/mL TD	13 (18.1%)	18 (10.5%)	
	>20 cp/mL	10 (13.9%)	22 (12.8%)	
HIV RNA 12 months pre-2DR switch, n (%):				0.30
	<20 cp/mL TND	58 (76.3%)	161 (84.7%)	
	<20 cp/mL TD	4 (5.4%)	10 (5.4%)	
	>20 cp/mL	14 (18.4%)	19 (9.9%)	
HIV RNA at 2DR switch, n (%):				0.15
	<20 cp/mL TND, n (%)	61 (80.3%)	167 (87.4%)	
	<20 cp/mL TD	8 (10.5%)	18 (9.4%)	
	>20 cp/mL	7 (9.2%)	6 (3.2%)	
*After 3TC/DTG Switch*				
HIV RNA 6 months post-2DR switch, n (%):				0.058
	<20 cp/mL TND	55 (72.4%)	164 (85.9%)	
	<20 cp/mL TD	10 (13.2%)	16 (8.4%)	
	>20 cp/mL	11 (14.5%)	11 (4.8%)	
HIV RNA 12 months post-2DR switch, n (%) *:				**0.004**
	<20 cp/mL TND	52 (69.3%)	154 (87.5%)	
	<20 cp/mL TD	13 (17.3%)	9 (5.1%)	
	Detectable > 20 cp/mL <50 cp/mL	6 (8%)	8 (4.5%)	
	Detectable > 50 cp/mL <200 cp/mL	4 (5.3%)	4 (5.3%)	
HIV RNA 24 months post-2DR switch, n (%) **:				**<0.0001**
	<20 cp/mL TND	50 (72.5%)	143 (89.9%)	
	<20 cp/mL TD	15 (21.7%)	6 (3.8%)	
	Detectable > 20 cp/mL <50 cp/mL	4 (5.8%)	6 (3.8%)	
	Detectable > 50 cp/mL <200 cp/mL	0 (0%)	4 (2.5%)	
HIV RNA 36 months post-2DR switch, n (%) ***:				**0.001**
	<20 cp/mL TND	30 (66.7%)	90 (92.8%)	
	<20 cp/mL TD	12 (26.7%)	5 (5.1%)	
	Detectable > 20 cp/mL <50 cp/mL	2 (4.5%)	2 (2.1%)	
	Detectable > 50 cp/mL <200 cp/mL	1 (2.2%)	0 (0%)	
Persistently TND during 24 months after switch °		34 (50%)	115 (74.2%)	**<0.0001**
Persistently TND during 36 months after switch °°		16 (18%)	73 (59.9%)	**<0.0001**

Characters in bold are statistically significant results. * data available for 251 pts; ** data available for 228 pts; *** data available for 142 pts; ° data available for 223 pts; °° data available for 176 pts. Abbr: 2DR: two-drug regimen; NRTI: Nucleoside Reverse Transcriptase Inhibitor; NNRTI: Non-Nucleoside Reverse Transcriptase Inhibitor; PI: Protease Inhibitor; INI: Integrase Inhibitor; 3TC: Lamivudine; DTG: Dolutegravir; TND: Target Not Detected; TD: Target Detected.

**Table 3:** Univariate and multivariate analysis for estimated factors with a predictive impact on HIV-RNA detectability after 36 months from the 2DR-3TC-containing switch.

	UNIVARIABLE		MULTIVARIABLE	
	OR (95% CI)	*p*-Value	OR (95%)	*p*-Value
Age,	1.01 (0.94–1.04)	0.098	1.01 (0.98–1.05)	0.25
Calendar year of HIV infection	0.97 (0.94–1.01)	0.21	0.99 (0.93–1.04)	0.78
Nadir CD4+ cell count	0.99 (0.98–0.99)	0.046	0.99 (0.99–1)	0.31
Previous HIV VR	2.10 (0.92–4.8)	0.077	2.0 (0.78–5.4)	0.14
HbcAb positivity	2.87 (1.58–5.2)	0.001	3.07 (1.39–6.79)	**0.005**

Abbr: OR: odds ratio; VF: Virological Rebound. Univariable odds ratios (ORs) associated with a lack of HIV RNA undetectability at T36 and their 95% confidence intervals (CIs) were calculated using logistic regression. A multivariable model was constructed in which risk factors with *p* < 0.1 in the univariable analysis were included in a full model, which were then excluded in a backwards-stepwise fashion if *p* < 0.05 using the likelihood ratio test. Characters in bold are statistically significant results.
